# Automated Counting of Airborne Asbestos Fibers by a High-Throughput Microscopy (HTM) Method

**DOI:** 10.3390/s110707231

**Published:** 2011-07-18

**Authors:** Myoung-Ock Cho, Seonghee Yoon, Hwataik Han, Jung Kyung Kim

**Affiliations:** 1 Department of Mechanical Engineering, Graduate School, Kookmin University, Seoul 136-702, Korea; E-Mails: myock.cho@gmail.com (M.-O.C.); zopy@naver.com (S.Y.); 2 School of Mechanical Engineering, Kookmin University, Seoul 136-702, Korea; E-Mail: hhan@kookmin.ac.kr; 3 Department of Integrative Biomedical Science and Engineering, Graduate School, Kookmin University, Seoul 136-702, Korea

**Keywords:** asbestos fibers, phase-contrast microscopy (PCM), high-throughput microscopy (HTM), automated counting, image processing and analysis

## Abstract

Inhalation of airborne asbestos causes serious health problems such as lung cancer and malignant mesothelioma. The phase-contrast microscopy (PCM) method has been widely used for estimating airborne asbestos concentrations because it does not require complicated processes or high-priced equipment. However, the PCM method is time-consuming and laborious as it is manually performed off-site by an expert. We have developed a high-throughput microscopy (HTM) method that can detect fibers distinguishable from other spherical particles in a sample slide by image processing both automatically and quantitatively. A set of parameters for processing and analysis of asbestos fiber images was adjusted for standard asbestos samples with known concentrations. We analyzed sample slides containing airborne asbestos fibers collected at 11 different workplaces following PCM and HTM methods, and found a reasonably good agreement in the asbestos concentration. Image acquisition synchronized with the movement of the robotic sample stages followed by an automated batch processing of a stack of sample images enabled us to count asbestos fibers with greatly reduced time and labors. HTM should be a potential alternative to conventional PCM, moving a step closer to realization of on-site monitoring of asbestos fibers in air.

## 1. Introduction

Asbestos has been widely used as a construction material due to its superior durability, heat resistance, and flame resistance. However, asbestos causes serious health problems, including lung cancer and malignant mesothelioma and it has been determined as a first-level carcinogen by the World Health Organization (WHO). The International Labor Office (ILO) has reported that more than 100,000 people around the world have died from exposure to asbestos [1]. Annually, about 3,000 Americans and 5,000 Europeans die from mesothelioma. Thousands of people are suffering from health damage due to a worldwide asbestos exposure epidemic. Thus, asbestos regulation is being actively pursued worldwide. The government of Japan had planned to reduce the use of asbestos in late 1980s in school environments. Subsequently, the Ministry of Health issued a total ban on asbestos use in 2005 [2]. The Korean government has also tightened its regulations on the use of asbestos, whereupon the recommended standard for asbestos exposure was adjusted into 0.1 fiber/cc by the Ministry of Labor in 2002. In 2004, the environment ministry raised the standard to 0.01 fiber/cc [3]. Eventually, asbestos-containing products and the use of asbestos were completely prohibited in 2009.

**Figure 1.**
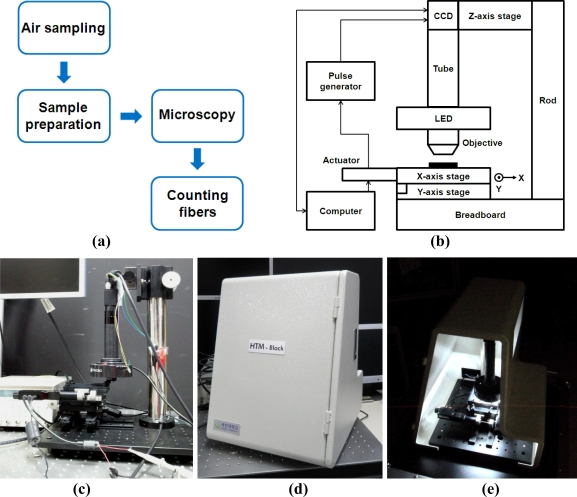
**(a)** Steps for conventional phase-contrast microscopy (PCM) method; **(b)** Schematic; and **(c)** photograph of setup for newly developed high-throughput microscopy (HTM) method; **(d)** Outside; and **(e)** inside views of HTM prototype system.

Accumulation of asbestos in the body by inhalation of airborne asbestos causes respiratory diseases as well as other virulent diseases. Unfortunately, asbestos related diseases are nearly impossible to treat. Thus, prevention entailing limited to no exposure to asbestos is the best way to reduce the incidence of the aforementioned diseases. Studies defining asbestos-containing materials are actively conducted as a means to prevent asbestos exposure. There are various methods for asbestos detection such as phase-contrast microscopy (PCM), polarized microscopy (PLM), X-ray diffraction method and electron microscopy (SEM and TEM). Although the PCM method is most widely used among these methods, it cannot clearly define certain types of asbestos including tiny asbestos fibers, and occasionally yields inaccurate results based on the subjective opinion of an operator. Figure 1(a) shows the PCM analysis procedure in which the air is collected through a filter and then a sample slide was prepared by dissolving the filter. Examiners count fibrous materials with lengths greater than 5 μm and aspect ratios over 3. Since the Walton-Beckett graticule, which is an eyepiece graticule specifically designed for asbestos fiber counting, was used, the field-of-view (FOV) in the PCM method is 0.00785 mm^2^. However, it is difficult to detect fibers shorter than 5 μm which comprise a large portion in the air. In addition, it is hard to resolve long fibers less than 0.25 μ m in diameter which are thought to be biologically active, due to the resolution limit of the phase contrast microscopy [4]. Also, it requires experts to define fibers. Total area being analyzed by one examiner in a day is less than 7.85 mm^2^ because one examiner observes under 100 FOVs on each slide, and it is hard to analyze more than 10 sample slides a day. Other methods including electron microscopy are known to increase detection accuracy. However, such methods still require trained experts and costly equipment [5]. Thus, new methods for detecting asbestos need to be developed, and some innovative techniques are being studied in many areas. We pay attention to asbestos detection methods through image analysis in particular.

The increased use of computers has enabled attempts at computerized image analysis. Inoue *et al.* [6] developed an Asbestos Fibers Automatic Counting System (AFACS) and verified its accuracy through comparison with the PCM method. They adopted a series of mathematical algorithms for image analysis such as “shading correction”, “thresholding”, “smoothing”, “border tracking”, “restoration of broken fibers”, “crossed and branched fiber processing” and “identification of asbestos fibers”. The fiber count obtained by the AFACS was equivalent to the median values of the manual counts produced at six different facilities. It is notable that less than 50% of the fibers were consistently counted by all counters, including the AFACS. As Baron mentioned in his review [7], there are many problems to be solved in image analysis method that arise from the complexity of fiber shapes including bundled and crossed fibers, focus drift of the fibers and messy backgrounds that include many other particles. Also, haloes around particles in the phase contrast image are often confused with fibers, and poor contrast between the fibers and background may obscure many fibers after thresholding.

Nevertheless, imaging techniques to automate the conventional manual counting method have been actively developed in recent studies. Kawabata *et al.* [8] developed a qualitative asbestos detection method in which image analysis modified conventional methods. Another promising method entails automatic counting of asbestos fibers. Image analysis easily detects asbestos fibers that are typically difficult to distinguish due to their small sizes or weak colors. Through image analysis, color contrasts can be controlled and exact size information can be determined [9]. Those techniques developed in Japan were based on the JIS standard. Furthermore, they investigated the polarized effects of asbestos by applying polarized light microscopy and X-ray diffraction method. Distinguishing asbestos from other particles was possible through the use of refraction phenomena of asbestos against light. Kawabata *et al.* [10] detected only asbestos fibers among many types of particles using both dispersion color and shape information. Moriguchi *et al.* [11] and Nomoto *et al.* [12] attempted to automatically count and detect asbestos using dispersion staining in which two images are matched. Then, color changes observed between the two images indicate the presence of asbestos. However, inconspicuous color changes still remained difficult to detect, thus requiring more time for detection and resulting in a more complicated process.

We have developed a High-Throughput Microscopy (HTM) method for automatic counting of asbestos fibers, which ultimately automated the conventional PCM method. We used three types of standard asbestos samples in order to observe asbestos fibers and to quantify fibrous particles in accordance with the concentrations. Since most problems in PCM are caused during the manual counting process, the results from HTM analysis based on automatic image processing are compared to PCM method. The feasibility and potential applications of HTM are also discussed.

## 2. Materials and Methods

### 2.1. Digital Microscope Setup with Robotic Stages

A 30 cm-long post was positioned on the breadboard and a CCD camera (IMB-20FT, imi tech) was fixed to the upper part of the post. We used a custom-made aluminum plate in order to align the CCD camera and the post in parallel. The camera was fastened to one side of the plate, and a z-axis stage (NT03-682, Edmond Optics) occupied the other side and was directly fixed to the post. The z-axis stage enabled precise focusing control through up and down movements. A body tube with a length of 160 mm was connected to the CCD camera and a 10x/0.25NA objective lens (NT36-132, Edmond Optics) was attached to the end of the body tube. A circular LED light was set up around the objective lens and its brightness was adjustable. Two linear stages (M-426A, Newport) were arranged under the objective in a stack in order to move in the *x* and *y* directions, respectively. The stages were connected to the linear actuators (T-NA08A50, Zaber Technologies) and automatically controlled by a software application (Zaber Console, Zaber Technologies). Rails were installed under the stage in the opposite direction to each other so as to readily find the initial position of the sample slide. Since the real available space was within 30 mm, with the exception of the stage-actuator connection area, rails were used to maximize the area in which images were obtained by moving it to the initial position of the samples. We also fabricated a black stage cover which conveniently fastened or released the sample slide, and reduced the background as well. Figure 1(b,c) shows the HTM setup. A custom-made HTM prototype system is shown in Figure 1(d,e).

### 2.2. Image Acquisition

We synchronized the robotic stages with the CCD camera in order to obtain many sample images at regular intervals for each move of the stage. Two actuators that were connected to each stage and controlled by a user-written program, were set up to move by 650 μm in the *x* and 490 μm in the *y* directions, respectively, throughout whole scanning area. The smallest distance that can be traveled by the actuator was 0.0476 μm. A TTL (Transistor Transistor Logic) signal triggered the CCD camera through a function generator (DG535, Stanford Research Systems) with a time delay of half a second in response to a typical pulse waveform generated at the movement of the actuator. After each stage completed its movement, a portion of sample slide image was acquired and stored through modified CamViewer software provided by the camera manufacturer. Each actuator traveled the same distance in *x* and *y* directions as the size of the CamViewer’s display window in order to capture the overall image of the sample slide. The resolution of the CCD camera was 1,600 × 1,200 pixels, and the size of the display window was 650 × 490 μ m^2^. One pixel corresponded to an area of 0.28 × 0.28 μ m^2^ when the 10× objective was used. The total number of steps of the actuator movement was determined by dividing the scanning area by the area of the display window. All movements were automatically controlled through the console program.

### 2.3. Image Processing

**Figure 2.**
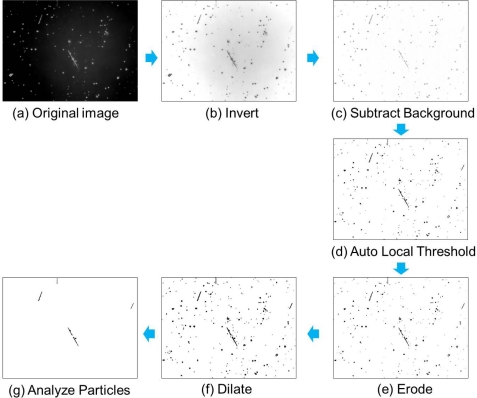
Asbestos sample images undergone specific steps for image processing and analysis in HTM method **(a)** Original image of amosite; **(b)** Invert; **(c)** Subtract Background (rolling = 10); **(d)** Auto Local Threshold (radius = 5); **(e)** Erode; **(f)** Dilate; **(g)** Analyze Particles (circularity = 0−0.33, size = 50–5,000).

Three-hundred images were captured for a sample slide by HTM setup; the total area was approximately 95.5 mm^2^. Therefore, analyzing a 120-fold larger area was possible in comparison to the conventional PCM method. We used an image processing and analysis program (ImageJ; http://imagej.nih.gov/ij/), and applied appropriate plugin menus offered by the software in order to detect asbestos. A stack of sample images was first processed by subtracting the background to produce the average background brightness over the image, and then corrected background illumination by “Auto Local Threshold” process. The threshold level was computed for each pixel according to the image characteristics within a window of radius “*r*” around it. The contrast of each image was maximized through additional processes such as “Smooth” to remove the noises and replace each pixel with the average of its 3 × 3 neighborhood, “Erode” and “Dilate” to subtract or add pixels to the edges of black objects. Then “Make Binary” process was applied to the image in order to convert to binary images. Lastly, the upper limit of the “circularity” was set to 0.33 and the “size” presented as pixels was set in the “Analyze Particles” process. The range for the circularity was calculated in order to count fibrous particles with aspect ratios greater than 3, in accordance to the counting rules used in the NIOSH 7400 method [13], and the criterion for the size was prescribed to exclude other particles with areas outside the range specified. Following these processes, small-sized or big clumps of dusts and circular-shaped patterns were eliminated. Figure 2 shows the image processing and analysis procedure for HTM method. Fiber density (fibers/mm^2^) or fiber concentration (fibers/cc) were calculated from the total fiber counts. Fiber concentrations detected by HTM method were then compared to those obtained by a professional asbestos analysis organization following PCM method.

### 2.4. Sample Slide Preparation

Three solid-phase standard asbestos samples, crysotile, amosite and crocidolite, from asbestos reference set (HSL037, Health and Safety Laboratory) were used for our validation study. Three grams of each asbestos sample were put into one liter of distilled water and crushed by ball milling for 30 min. Subsequently, the resulting samples were dispersed by an ultrasonic wave. A drop of surfactant (Triton X-100, Sigma-Aldrich) was added to each sample to prevent agglomeration of the fibers. We made a sample slide in which two pieces of 50 μm-thick double-sided tapes were attached to a 75 × 25 mm^2^ glass slide in parallel and then an 18 × 18 mm^2^ coverslip was placed on it. Twenty microliters of serially diluted asbestos sample solutions were injected into the gap between the slide and the coverslip, and the gap was sealed with nail polish [14]. By applying the HTM method, the entire area of the coverslip was scanned and fibrous particles satisfying the prescribed criteria were counted through appropriate image processing and analysis procedure. We obtained manual count of fibers in the asbestos sample with known concentration and regarded it as a control. Then we determined a set of parameters as described in the previous section for image analysis which produced the best approximate result with the manual count. The optimized set of parameters was applied to a stack of images using a macro file. To evaluate the accuracy of HTM in comparison with PCM, we also analyzed five PAT (Proficiency Analytical Testing) standard asbestos samples and 11 airborne asbestos samples collected on-site. The PAT samples are quality control standard samples used to verify the ability of the analysis for harmful materials in the US NIOSH PAT program. Sample slides containing the PAT and the on-site samples and corresponding PCM analysis data were kindly offered by the Korean Industrial Health Association.

## 3. Results and Discussion

We have automated the conventional PCM method primarily used for estimating asbestos particle concentrations in air. The typical time required for the PCM analysis is about 90 min in observing 100 field-of-views and can be varied substantially depending on the operator’s degree of skill, the amount of fibers in the sample slide and the level of the background noise. The newly developed automatic counting method, HTM, provides notable advantages in comparison to PCM. In particular, HTM can analyze the whole area of the asbestos sample slide considerably fast reducing time consumption. The stages move every second and the images are captured simultaneously. Therefore, it takes only 5 min to obtain 300 images and the analysis of the whole images is completed in a few minutes by a batch process executing a list of commands.

**Figure 3.**
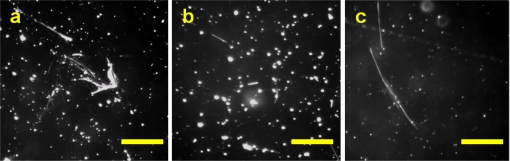
Raw images of ball-mill ground asbestos samples obtained by HTM. **(a)** chrysotile; **(b)** amosite; **(c)** crocidolite (scale bar = 100 μm).

Prior to setting the control parameters for the stage movement, we confirmed whether the traveled distance of the objects on the display window was the same as the stage. Evenly spaced grid lines at intervals of 650 μm in the *x* and 490 μm in the *y* directions were drawn on a paper, and then it was attached on a slide. The lines passed through the display window one by one without leaving unscanned area when the stage moved at regular intervals. We also compared FOVs obtained by the 10× objective in HTM and the 40× objective in PCM. The area of the microscope’s FOV was about 0.237 mm^2^ and the diameter was 550 μm. The image acquired by the CCD camera through the 10× objective possessed a 650 × 490 μm^2^ rectangular shape and its area was approximately 0.318 mm^2^, which was slightly larger than the FOV of PCM. We used the solid-phase standard asbestos samples in order to evaluate the performance of HTM and to establish proper criteria for image processing and analysis. Ball-mill ground asbestos samples were serially diluted to have different concentrations and then the total number of fibers at each concentration was counted by both manual and HTM methods. Figure 3 shows the raw images of an asbestos sample slide typically obtained by HTM.

We captured 300 images at each concentration of asbestos samples, and analyzed 100 images at each concentration level by running the macro file which carried out a series of commands consecutively to apply the set of parameters for image processing and analysis.

**Figure 4.**
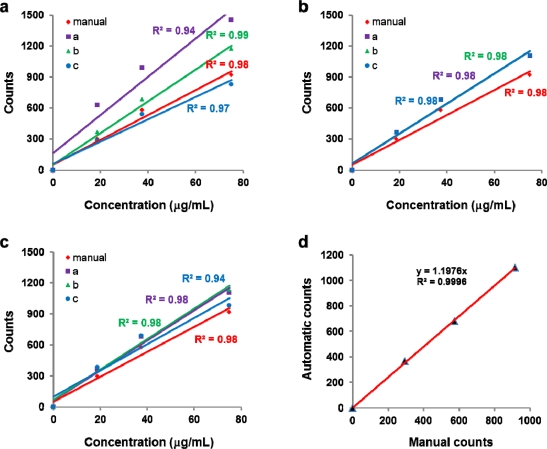
Total fiber counts *versus* asbestos concentration affected by change in the parameter for **(a)** “Auto Local Threshold” process (radius: a = 2, b = 5, c = 10); **(b)** “Threshold” process ({min, max}: a = {50, 170}, b = {0, 40}, c = {30, 255}): the changes in the threshold value are insignificant and a, b and c are completely overlapped; and **(c)** “Analyze Particles” process (size: a = 50–5,000, b = 10–5,000, c = 10–10,000); **(d)** Correlation between manual counts and automatic counts from HTM analysis with respect to the asbestos concentration.

Figure 4 demonstrates the impact of the individual parameter for image analysis on the total fiber counts with only one parameter varied at a time. There was relatively high deviation increasing with the asbestos concentration in the total fiber counts with respect to the parameter value “*r*” (*r* = 2, 5, 10) for the “Auto Local Threshold” process. In the case of *r* = 10, there was a best correlation with the manual count [Figure 4(a)]. Changes in the threshold value ({min, max} = {50, 170}, {0, 40}, {30, 255}) for further removing the background afterwards were insignificant [Figure 4(b)]. After separating the fiber images from the background, the size criterion for the “Analyze Particles” process should be carefully set to secure the counting accuracy. There was a little change in the total fiber counts with variation in “size” value (size = 50–5,000, 10–5,000, 10–10,000) [Figure 4(c)]. Among nine parameter sets obtained from the combinations of the parameters “*r*” and “size” (see supplementary information Figure S1), the most suitable parameter set was deduced: Subtract Background, rolling = 10; Auto Local Threshold, *r* = 5; Smooth; Threshold, {min, max} = {50, 170}; Make Binary; Dilate; Erode; Analyze Particles, size = 50–5,000, circularity = 0–0.33. There was a remarkably high correlation in the total fiber counts obtained by manual counting and HTM analysis with the best parameter set as shown in Figure 4(d).

**Figure 5.**
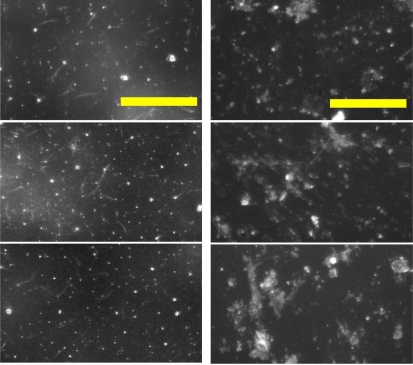
Raw images of asbestos samples obtained by HTM. Left column: PAT standard samples, Right column: on-site airborne samples (scale bar = 100 μm)

Figure 5 shows the raw images of the PAT standard asbestos samples and on-site asbestos samples collected in air. The level of background noise for the PAT standard sample images was similar with the ball-mill ground sample images, but much higher for the on-site sample images. We applied the optimized set of image processing parameters described above, considering the background level of each sample for HTM analysis. Comparisons of HTM and PCM analyses for the PAT standard samples and the on-site samples are displayed in Table 1 and Table 2, respectively. The PAT sample has LCL (Lower 95% Confidence Limit) and UCL (Upper 95% Confidence Limit) for the fiber density provided by statistical calculations. The fiber density of only one sample (No. 177-2) out of five was slightly exceeded UCL by 10% (Table 1). The fiber concentrations measured by HTM analyses of the on-site samples from 11 different workplaces approached fairly well to those from PCM analyses (Table 2). Discrepancies for the samples collected at E-1 and E-2 were extraordinarily higher than the other samples, which attributed mainly to darker and unclean background in the asbestos sample images. A different set of image processing parameters should reduce these discrepancies. When we set the parameters rolling = 20 for “Subtract Background” and *r* = 10 for “Auto Local Threshold”, for instance, the HTM result for E-1 sample was 0.013 fibers/cc comparable to the PCM result (see supplementary information Figure S2). Further study is needed to implement the function of automatic adjustment of the parameters considering the background noise level of the asbestos sample image.

In addition, the procedure needs to be enhanced by implementing the counting rules [13] described in NIOSH 7400 in order to treat crossed fibers and fiber bundles, which were not considered thoroughly at this time. Furthermore, more PAT standard samples should be analyzed to assure the quality of HTM analysis.

The proposed HTM method can detect fibrous particles, but cannot differentiate between asbestos and other fibers. Kuroda *et al.* [5] and Ishida *et al.* [15] proposed a novel method for staining the airborne asbestos fibers using asbestos-adhesive proteins, such as DksA and GatZ, and demonstrated the feasibility of selective detection of each type of asbestos. We are currently improving the sensitivity and selectivity of HTM method by using a sample slide pre-treated with the asbestos-adhesive proteins bound only to asbestos fibers [16]. Our HTM method should be a useful tool for on-site monitoring of airborne asbestos concentration. Furthermore, the application of the HTM system can be extended to laboratory-scale robotic screening processes in the biomedical field, such as malaria diagnosis, rare cell detection and bacterial colony counting.

## 4. Conclusions

We have developed an automatic counting method for airborne asbestos fibers, which acts as an alternative to the conventional phase-contrast microscopy (PCM). We suggested a high-throughput microscopy (HTM) method using a digital CCD camera running synchronously with a 2-axis robotic stage. Hundreds of images acquired by scanning an asbestos sample slide automatically were analyzed by a specifically-designed image processing algorithm to enumerate bright fibrous particles. Our HTM method can provide shape information and the concentration of asbestos fibers in air. Also, a large amount of image data was processed successfully in a short period of time through HTM, significantly reducing the time consumption, hard labors and variability inevitably accompanied with PCM.

## Supplementary Information



## Figures and Tables

**Table 1.** Comparison of fiber density measured by PCM and HTM for PAT standard asbestos samples.

**No.**	**PCM (fiber/mm^2^)**	**HTM (fiber/mm^2^)**	**LCL (Lower 95% Confidence Limit)**	**UCL (Upper 95% Confidence Limit)**
177-2	316	587	155	535
179-1	305	333	149	515
180-3	439	483	280	633
181-3	255	407	128	426
182-1	406	365	211	664

**Table 2.** Comparison of fiber concentration measured by PCM and HTM for on-site airborne asbestos samples.

**Workplace**	**Air volume sampled (L)**	**PCM (fiber/cc)**	**HTM (fiber/cc)**	$\frac{\left|\text{PCM}-\text{HYM}\right|}{\text{PCM}}\times 100\%$
L	252	0.011	0.0109	0.909
K	252	0.014	0.0162	15.7
C-1	140	0.033	0.0361	9.39
C-2	140	0.025	0.0277	10.8
J-1	242	0.050	0.0333	33.4
J-2	242	0.080	0.05	37.5
S	720	0.029	0.018	37.9
E-1	242	0.020	0.051	155
E-2	242	0.020	0.038	90
B-1	240	0.040	0.027	32.5
B-2	240	0.050	0.0376	24.8
