# Supplementary material for: Automated Counting of Airborne Asbestos Fibers by a High-Throughput Microscopy (HTM) Method
Source: Sensors (Basel). 2011 Jul 18;11(7):7231–42. doi: 10.3390/s110707231 (PMC3231659; doi:10.3390/s110707231)

Supplementary Information

Figure S1. Effects of changes in the parameters '*r*' for 'Auto Local Threshold' process and size for 'Analyze Particles' process on the correlation between manual counts and automatic counts from HTM analysis with respect to the asbestos concentration. The parameter for 'Threshold' process was fixed at {min, max} = {50, 170}. (a) $r = 2$, size = 50–5,000, (b) $r = 2$, size = 10–5,000, (c) $r = 2$, size = 10–10,000, (d) $r = 5$, size = 50–5,000, (e) $r = 5$, size = 10–5,000, (f) $r = 5$, size = 10–10,000, (g) $r = 10$, size = 50–5,000, (h) $r = 10$, size = 10–5,000, (i) $r = 10$, size = 10–10,000

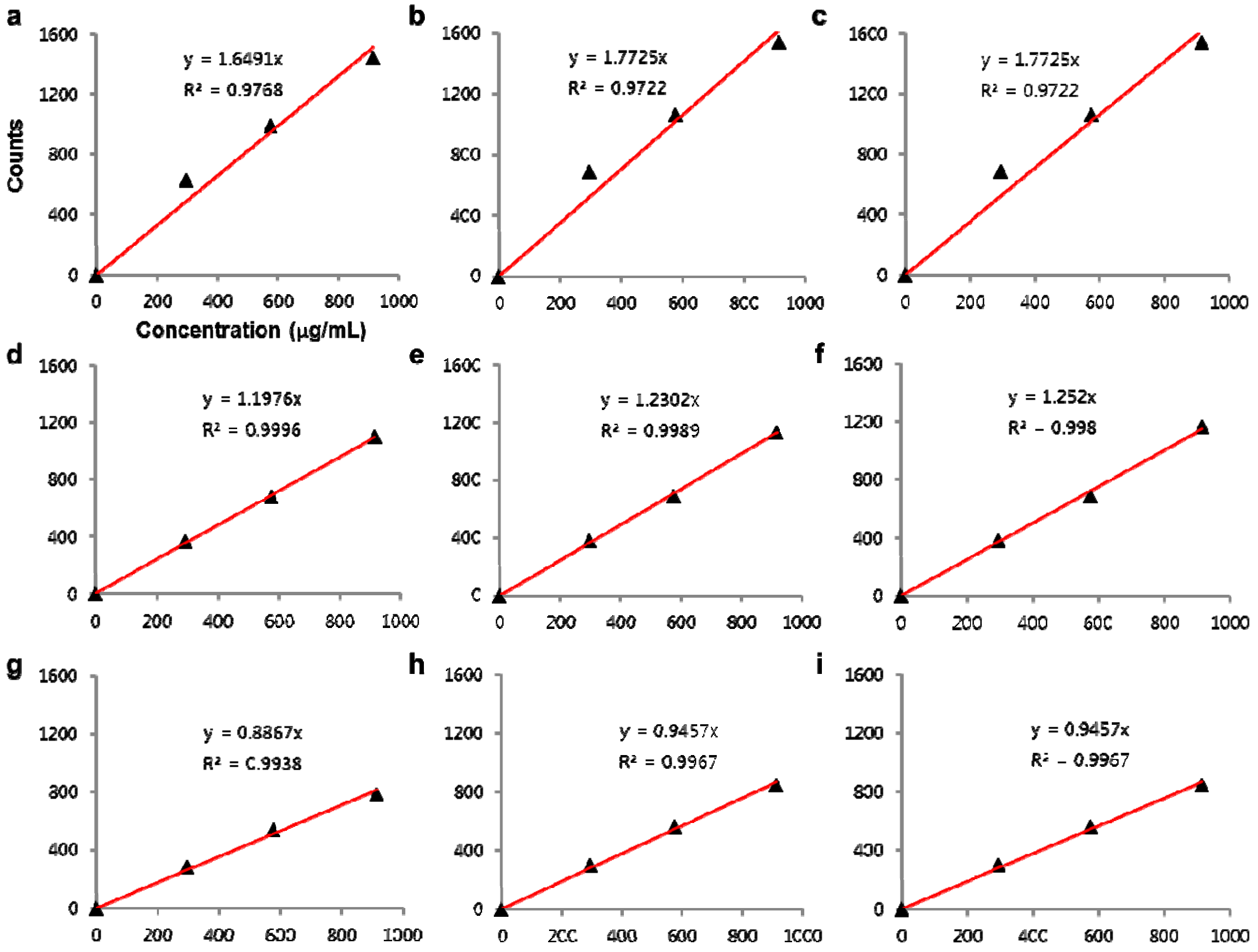

Figure S2. Effects of changes in the parameters ‘rolling’ and ‘radius’ for E-1 and E-2 samples.

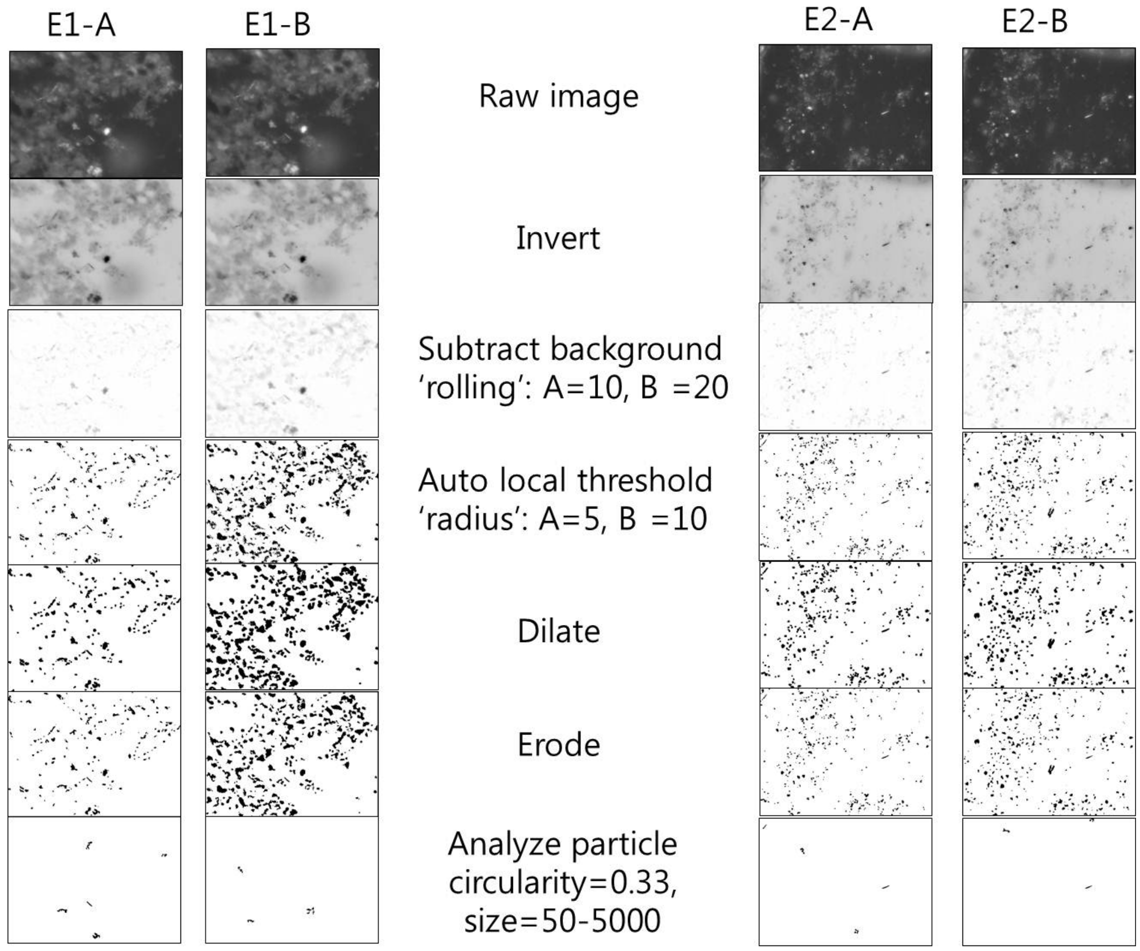

Supplement: Supplementary file 1 [file sensors-11-07231-s001.pdf]
